# Improved and Highly Reproducible Synthesis of Methacrylated Hyaluronic Acid with Tailored Degrees of Substitution

**DOI:** 10.1021/acsomega.4c00372

**Published:** 2024-06-06

**Authors:** Marta Pérez-Lloret, Andrea Erxleben

**Affiliations:** School of Biological and Chemical Sciences, University of Galway, Galway H91 TK33, Ireland

## Abstract

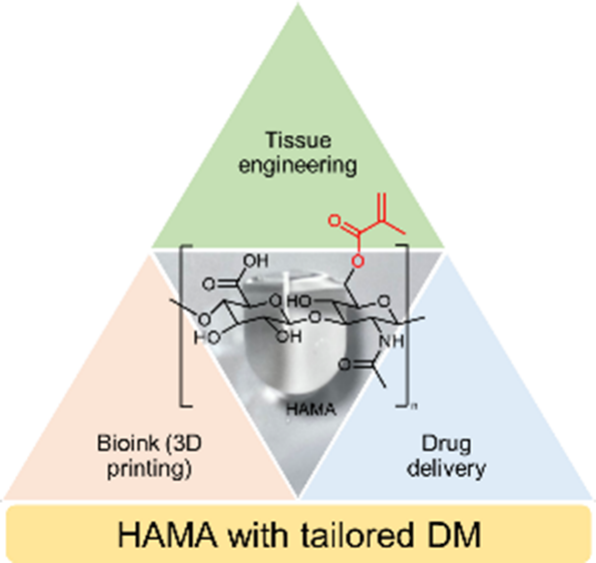

Methacrylated hyaluronic acid (HAMA) is a versatile material that has gained significant attention in various pharmaceutical and biomedical applications. This biocompatible material can be photo-cross-linked in the presence of Irgacure 2959 (I2959) to produce hydrogels. Controlling the degree of methacrylation (DM) is crucial since it plays a pivotal role in determining the properties and thus the potential applications of the gels. We report herein a new green approach for the highly controlled and tailored modification of hyaluronic acid (HA) with methacrylic anhydride (MA). The reaction conditions of previously reported procedures were optimized, leading to a decreased reaction time (3 h instead of 24 h) and consumption of fewer equivalents of MA (5 equiv instead of 20) and water as the sole solvent. By changing the amount of base added, HAMA with three different DMs was obtained: 19, 35, and 60%. The influence of the molecular weight of HA, degree of substitution, and concentration of the HAMA solution prior to photo-cross-linking on the rheological, swelling, and degradation properties of HAMA hydrogels was also studied in this work.

## 1 Introduction

Materials based on hyaluronic acid (HA) are versatile biomaterials that have attracted significant attention in the fields of tissue engineering, drug delivery, cell encapsulation, and regenerative medicine.^1,2^ While displaying significant differences from the native polymer, functionalized HA derivatives are biocompatible, biodegradable, and able to mimic the extracellular matrix of native tissues.^3−5^

HA has a primary alcohol and a carboxyl group which can be exploited as anchoring points for functionalization.^3,5,6^ One of the most used HA derivatives is methacrylated HA (HAMA). Obtained by esterification of HA with methacrylic anhydride (MA) to introduce methacrylate groups along the HA backbone, it can be used to cross-link the polymer through chemical cross-linking^7^ or photopolymerization.^8^

HAMA has been widely studied for its properties for therapeutic purposes. It can be used as a hydrogel for osteoarthritis treatment,^9^ to promote wound healing,^10^ and as a tissue adhesive.^11^ HAMA-based hydrogels have been demonstrated to support the growth and differentiation of various cell types, including chondrocytes, stem cells, and fibroblasts.^5^ Another important application of HAMA is in the field of drug delivery, with a special focus on the delivery of cancer drugs. HA is the primary CD44 binding molecule, a receptor overexpressed in a variety of tumors.^12^ Many recent studies have focused on exploiting the properties of HA to develop nanocarriers that demonstrate preferential tumor accumulation and increased cellular uptake,^13^ reaching in some cases clinical trials.^14^ Various stimuli-responsive HAMA hydrogels have been developed in the past years, including electro-responsive hydrogels for ibuprofen release,^15^ pH-sensitive^16^ and enzyme-sensitive^17^ nanohydrogels releasing doxorubicin, curcumin-loaded nanoparticles,^18^ and microneedles for the transdermal delivery of doxorubicin.^19^

Due to its unique properties in terms of biocompatibility and water retention, this polymer has emerged as a very promising material in the fields of tissue engineering, regenerative medicine, and bioink for 3D printing.^20−23^ Because HA is a native component of cartilage, and its regeneration is still a challenge nowadays, multiple studies have been conducted in this domain.^24^ HAMA gels were reported to successfully promote mesenchymal stem cell chondrogenesis^25^ and can also be exploited as synthetic extracellular matrix analogues.^26^ Some recent studies showed that HAMA scaffolds are capable of promoting osteogenesis of human mesenchymal stem cells,^27^ demonstrating this type of material could act as a carrier to deliver multipotent stromal cells to the site of injury.^28^

HA with different molecular weights is commercially available, which has an influence on its biological effects. Very-low molecular weight HA (<4 kDa) presents nonapoptotic properties and acts as an inducer of heat shock proteins, while low molecular weight HA (6–20 kDa) has angiogenic, phlogotic, and immunostimulatory activities. Medium molecular weight HA (20–200 kDa) is involved in biological processes, such as wound healing, ovulation, and embryonic development. Finally, high molecular weight HA (>500 kDa) is of interest for cartilage regeneration, as a space filler, a natural immunologic depressant, and an antiangiogenic.^29,30^

The physicochemical properties of HAMA gels, such as mechanical stress resistance, swelling behavior under physiological conditions, and degradation rate, are determined by the cross-linking density. Consequently, by selecting the molecular weight and adjusting the cross-linking density, a material with tuned properties can be produced. The degree of cross-linking depends on the concentration of HAMA in the cross-linking solution and the number of methacrylate groups. Therefore, robust and scalable protocols for the synthesis of HAMA with a well-defined, adjustable degree of methacrylation (DM) are crucial to tailoring the properties of HAMA to different applications. However, despite the wide use of HAMA in biomedical research, synthesizing HAMA with a specific and reproducible DM has remained a challenge and presents an obstacle to clinical translation. In particular, the problem of reproducibility of the DM in the context of Good Manufacturing Practices has been discussed in the literature.^31^ A widely used procedure involves the reaction of HA with MA in aqueous solution on ice with a reaction time of 24 h.^7,8^ Careful control of the pH value between 8 and 10 is required to neutralize the formed methacrylic acid and to push the reaction forward. Although a 20-fold excess of MA is typically used, the DM is generally low, often <30%, mainly due to the low solubility and hydrolysis of MA in water.^7,8^ Hachet et al. published a procedure that gave DMs in the 10–50% range but required the use of (toxic) dimethylformamide as a cosolvent.^26^ Oudshoorn et al. used glycidyl methacrylate as the reagent and performed the reaction in dry DMSO in the presence of 4-dimethylaminopyridine at 60 °C for 24 h.^32^ There is only one report in which DMs of 88 and 160% were achieved in aqueous solution by applying 10 and 20 equiv of MA, respectively.^33^ By contrast, Spearman et al. used a 20- and 40-fold excess of MA and reported DMs of 53 and 86% in water.^21^

These unsatisfactory data on the DM and conflicting reports suggest that there is a need for a systematic study and improved synthesis for HAMA with a high and controllable DM. Therefore, the aims of the present study were (1) to perform a comprehensive optimization and investigation of the relationship between the reaction conditions and the DM. Specifically, we systematically varied the way of pH control and MA addition in a total of 100 screening experiments; (2) to improve the efficiency of the methacrylation reaction while applying “green” principles, i.e., reducing the reaction time, reducing the consumption of MA by minimizing loss due to hydrolysis and achieving high DMs without the use of organic cosolvents or catalysts; and (3) to evaluate the rheological properties, swelling behavior, and enzymatic degradation rate of the hydrogels obtained by the optimized method.

## 2 Experimental Section

### 2.1 Material

HA (8–15, 40–50, 80–100, 200–500, and 600–1000 kDa) was purchased from Biosynth. 2-Hydroxy-4′-(2-hydroxyethoxy)-2-methylpropiophenone (Irgacure 2959, I2959) and MA were obtained from TCI. Hyaluronidase from bovine testes type I–S (lyophilized powder, 400–1000 units/mg), carbazole, sodium tetraborate decahydrate, and glucuronic acid were obtained from Sigma-Aldrich. Phosphate-buffered saline (PBS) tablets (Dulbecco A, 150 mM, pH 7.3), sodium carbonate, sodium bicarbonate, and concentrated sulfuric acid (95%) were purchased from Fisher Scientific. Ethanol and dimethyl sulfoxide (laboratory reagent grade), and Milli-Q water were used in all the preparations.

### 2.2 Instrumentation

Dynamic rheology measurements were performed on an Anton Paar modular compact rheometer (MCR 302) with a parallel plate setup with a 24 mm plate geometry at 1 mm distance. Experiments were performed on gels of 1 mm thickness at 20 °C in triplicate. The samples were subjected to a frequency sweep from 0.1 to 100 Hz with a constant strain of 10%, and an amplitude sweep at constant 1 Hz frequency of oscillation and constant strain of 1%. The strain was selected based on published stress vs strain curves of HAMA hydrogels that showed a linear behavior at strains <20%.^8^

Ultraviolet–visible (UV–vis) spectra were recorded using a Varian (Cary 100) UV–vis spectrometer.

NMR spectra were recorded on an Agilent 600 MHz spectrometer (Agilent Technologies, Santa Clara, CA, USA) equipped with a 5 mm CryoProbe and on a Varian Innova 500 MHz spectrometer (Varian, Palo Alto, CA, USA) with a 5 mm OneNMR probe. All the spectra were measured in D_2_O using the residual solvent peak as the reference.

### 2.3 Screening of the Reaction Conditions for HA Methacrylation

Screening reactions were performed on a small and large scale. Small-scale reactions were done in 4 mL screw cap vials using 20 or 30 mg of HA and variable volumes of MA and NaOH (1 M) under different conditions. For a larger scale, 250 mg of HA was dissolved in 25 mL of solvent in a 50 mL round-bottom flask. All the reactions were performed in the dark. Purification was achieved by precipitation with cold ethanol, and the products were recovered either by centrifugation or filtration.

### 2.4 Optimized HAMA Synthesis

HA was dissolved in distilled H_2_O at 1% weight concentration under magnetic stirring at 4 °C, and the pH was corrected to 8 with 1 M NaOH. After adding 5 equiv of MA under dark conditions, a certain volume of 1 M NaOH (0.5, 1, or 2 equiv per addition) was added every 30 min, 4 additions in total. Thirty minutes after the last addition, the sample was precipitated in cold ethanol, and the solid was recovered either by centrifugation or filtration with a PC membrane filter. The precipitate was washed with small portions of ethanol, dried at 40 °C and kept protected from light in the fridge. The DM was determined by ^1^H NMR spectroscopy from the relative intensities of the NHC(O)CH_3_ signal (3H, singlet, 2.03 ppm) and the signal of the methacrylate protons at 6.19 and 5.75 ppm.

### 2.5 Swelling Behavior

The degree of swelling was performed as previously reported with some modifications.^32^ Briefly, hydrogels were prepared by pipetting 200 μL of HAMA solution (0.5–3% wt in PBS) containing I2959 (0.3% wt) into round-bottom 96 well plates. The mixture was photo-cross-linked by irradiation at 365 nm for 10 min (55 mW/cm^2^). After irradiation, the gels were carefully weighted (*W*_0_) and transferred to a vial containing 2 mL of PBS. The samples were incubated for 24 h in the dark at room temperature, and their mass was recorded (*W*_24_). All the samples were prepared in triplicate. The swelling degree is calculated as^34^
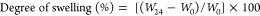


### 2.6 In Vitro Gel Degradation

In vitro degradation of HAMA hydrogels was followed by the carbazole assay.^35^ Hydrogels were prepared by pipetting 60 μL of HAMA solution (0.5–3% wt) in PBS containing I2959 (0.3 wt %) in round-bottom 96 well plates. Upon photo-cross-linking (10 min, 365 nm, 55 mW/cm^2^), the gels were retrieved with a spatula, weighed, and transferred to a vial for incubation under stirring at 37 °C in 2 mL of PBS containing 100 U/mL of hyaluronidase (Hyal). At predetermined times, 100 μL aliquots of supernatant were taken and replaced with 100 μL of fresh PBS with 100 U/mL of Hyal. The aliquots were added to 1.5 mL of 0.025 M sodium tetraborate in H_2_SO_4_, shaken, boiled in a water bath for 10 min, and cooled to room temperature. Then, 100 μL of carbazole solution (0.125% wt in ethanol) was added, and samples were shaken and boiled for 15 min. After cooling to room temperature, the UV–visible absorption spectra were recorded against a blank sample. The uronic acid content was determined using solutions of known concentrations of glucuronic acid as a standard to build a calibration curve in the same concentration range as the degradation samples.

## 3 Results and Discussion

### 3.1 HAMA Methacrylation

**Figure 1:**
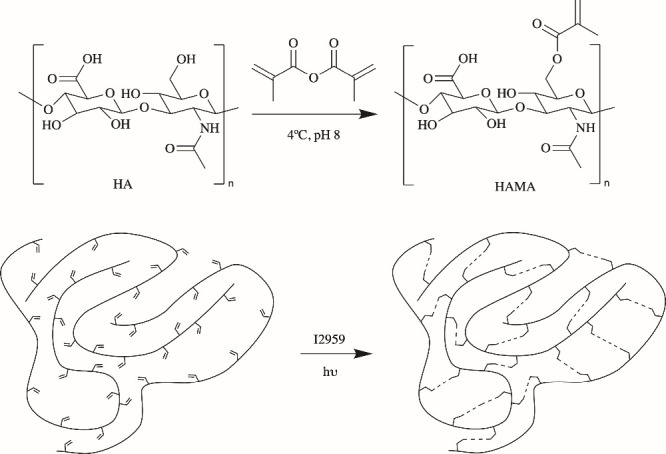
HA methacrylation and photo-cross-linking with I2959.

The most frequently used literature protocol for the synthesis of HAMA is as follows^7,8^: a 20-fold excess of MA is added to a 1% weight solution of HA in deionized water. The pH is then adjusted to 8 with 5 M NaOH, and the reaction is performed for 24 h on ice (Figure 1). Our attempts to reproduce the procedure led to inconsistent results in the DM. Some variations found in the literature include correcting the pH during the reaction; however, no details about the frequency were mentioned.^20,21,31,36^ During our first attempts to reproduce the reaction, some observations were noted. First, the mixture was found to be acidic prior to the pH correction all the time; second, the number of times the pH was corrected had a direct influence on the final DM, and finally, the miscibility of MA with water is very poor.

It is well-known that esterification reactions are pH- dependent, and considering this particular reaction has methacrylic acid as a by-product, it will explain the first and second observations. In an attempt to improve the DM, stirring was replaced with sonication, but the results were extremely inconsistent, and this approach was immediately discarded. To obtain a better understanding of the factors influencing the reaction, several conditions were investigated. The solvents, reaction times, temperature, equivalents, and type of additions tested are summarized in Table 1, and full details of the screening experiments are provided in the Supporting Information (Tables S1–S6).

**Table 1:** Summary of the Variables in the Screening of the Reaction Conditions for the Methacrylation of HA

solvent	MA eq	NaOH eq	pH adjustment
H_2_O	0.5	0.5	none
PBS buffer	1	1	before MA addition
CB^a^ buffer	2	2	titration with NaOH
DMSO	5	4	
DMSO/H_2_O (1:1)	8.3	8	
	10		

time	temperature	MA addition	time between additions
2–6 h^b^	ice bath	one addition	15 min
24 h	RT	titration	30 min
	30 °C		

a Carbonate-bicarbonate buffer (CB) pH 8.5.

b Total time depends on the number and time between additions.

The first set of screening experiments was performed to understand the influence of the pH, solvent, and temperature on the reaction (Table S1). In the experiments with a reaction time of 24 h, the pH was corrected only once. This led to low DMs, while the samples that had their pH corrected several times displayed higher degrees of substitution. Strangely, it was noticed that using buffers (PBS pH 7.3 or carbonate-bicarbonate pH 8.5) gave a lower DM than water alone. This result can be explained by the fact that acid anhydrides are hydrolyzed under basic conditions. A literature review showed that similar polymers have been methacrylated using alternative strategies. In their recent work, Lee et al.^37^ proposed a novel approach involving sequential time-lapse loading to prepare methacrylated gelatin. Following the same strategy, we tested the stepwise addition of small portions of MA to HA. In order to investigate if MA degradation could be mitigated and a higher DM could be achieved, 5 equiv of MA were added every 30 min, 6 additions in total. Before each addition, the pH value was corrected. This led indeed to a higher DM (Table S1). Using DMSO or DMSO/H_2_O as the solvent gave low DMs and was not further pursued.

To confirm the early results obtained, a second set of experiments was designed. Parameters such as buffer, temperature, time between additions, and equivalents of MA were varied. In this set of experiments, MA was always added in small portions. As observed in Table S2, a low temperature seemed to affect the degree of methacrylation in most cases positively. Increasing the time between additions from 30 min to 1 h did not clearly affect the DM. In addition, the use of buffers did not give a clear trend.

**Figure 2:**
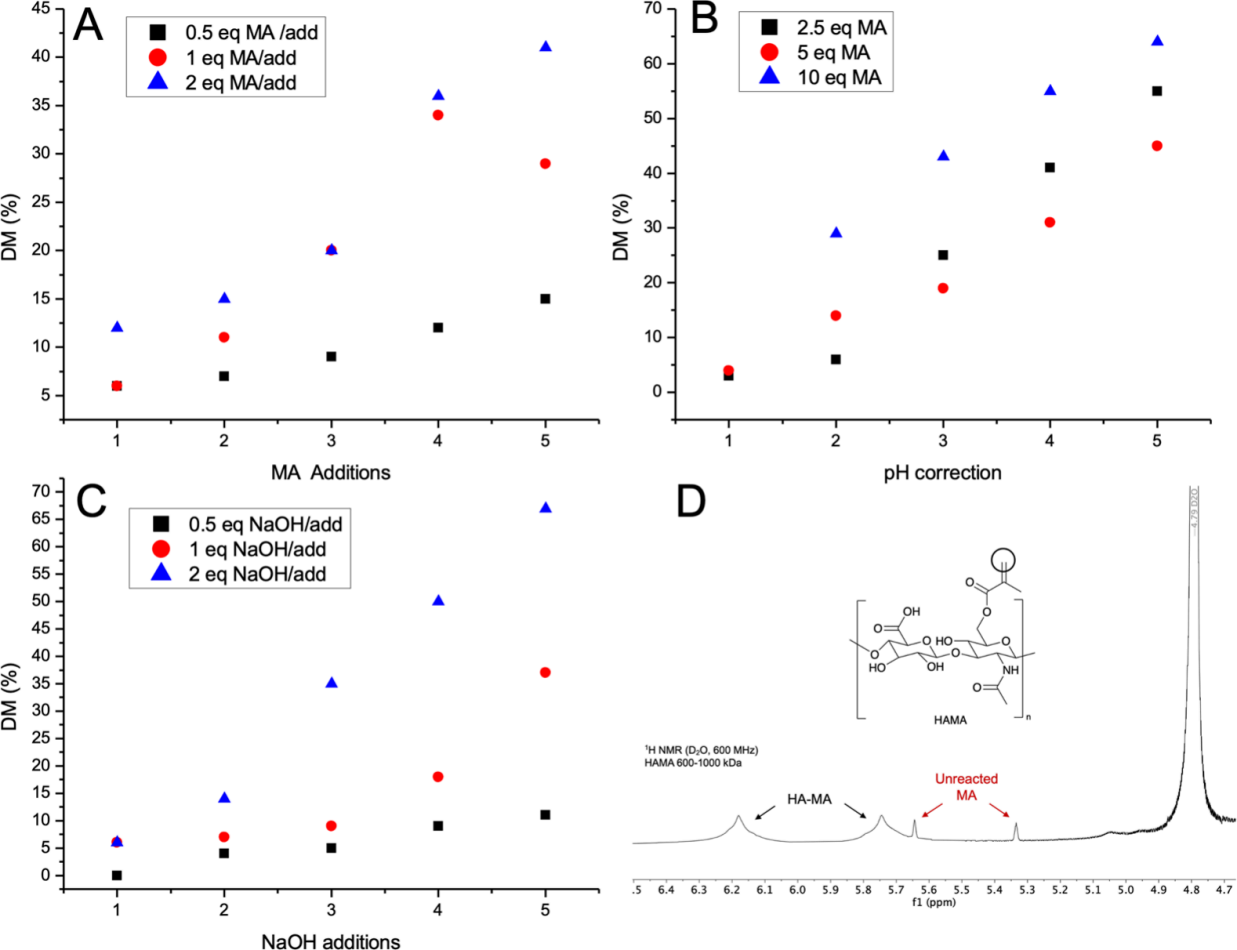
Effect of the different titration strategies on the final DM. 250 mg of HA (80–100 kDa) was dissolved in 25 mL of water and reacted at 4 °C under stirring in the dark. (A) MA (0.5, 1, and 2 equiv) was added every 30 min, and the pH value was corrected prior to each addition; (B) MA was added (2.5, 5, and 10 equiv), and the pH value was corrected every 30 min; (C) MA was added (2.5, 5, and 10 equiv), and NaOH (1 M; 0.5, 1, and 2 equiv) was further added every 30 min. (D) Determination of the DM by ^1^H NMR spectroscopy (D_2_O, 600 MHz).

The effect of the MA “titration” strategy was further investigated with a new type of screening experiment. The scale of the reaction was increased to 250 mg, and prior to the pH correction and subsequent MA addition, a small aliquot was taken and analyzed by ^1^H NMR spectroscopy. As shown in plots (A) and (B) of Figure 2, pH correction displayed a better linear correlation with the DM than did titration with MA. To confirm the hypothesis, 5 additions of 0.5, 1, and 2 equiv of NaOH (1 M) replaced the pH correction in experiment (C), while the rest of the conditions were unaltered. The results illustrated in Figure 2C suggested that the additions of NaOH and the number of equivalents have a direct influence on the final degree of substitution.

A new set of experiments was designed to evaluate the effects of the molecular weight of HA, temperature, reaction time, equivalents of MA, and the NaOH titration strategy (Tables S3–S6). It was concluded that

1. Under the same conditions, 4 equiv of MA are the minimum excess required to be able to tune the final degree of substitution only by the influence of the NaOH titrations. A higher fold excess has a low or no effect on the final DM.
2. Reactions performed on ice lead to a higher DM than reactions performed at room temperature.
3. The molecular weight of HA does not seem to influence the final DM.
4. Titrations with NaOH every 30 min appear to be optimal. Less time (15 min, see Tables S2 and S6) led to a lower DM (insufficient reaction time), while longer times (1 h) did not increase the DM. Most likely after 30 min, the pH value that decreases during the reaction reaches too low a value so that the reaction does not proceed further.
5. The number of titrations and NaOH equivalents have a direct influence on the final DM.

Based on these conclusions, we propose a new method for HAMA preparation. The reaction, as described in the experimental section, was tested for reproducibility purposes on low, medium, and high molecular weight HA in batches ranging from 200 mg up to 3 g. As illustrated in Table 2, this new approach led to tailored and highly reproducible DMs which can be predefined by adjusting the equivalents of NaOH added.

**Table 2:** Summary of the DM Obtained during the New Methacrylation Approach Performed with HA of Different Molecular Weight (8–15, 40–50, 80–100, 200–500, and 600–1000 kDa) and Batch Sizes Ranging from 200 mg up to 3 g

NaOH eq	DM %	number of replicates
0.5	19.2 ± 1.0	10
1	35.3 ± 1.0	11
2	60.1 ± 0.9	10

The addition of photo-cross-linkable methacrylate groups is described for various (bio)polymers in the literature including other polysaccharides (e.g., chitosan,^38^ alginate,^39^ and dextran^40^) and proteins (gelatin^37^ and silk fibroin^41^). While this work focuses on hyaluronic acid, we anticipate that our “tritation strategy” is also applicable to the optimization of the methacrylation of other polycarbohydrates. In the case of proteins, methacrylation takes place at the free amino groups of lysine residues. Careful control of the pH is even more crucial here, as the amino groups must be deprotonated to be reactive toward MA.^37^ Furthermore, the protein may precipitate when the pH value reaches the isoelectric point. Therefore, the application of our method to protein-based hydrogels or polysaccharides containing primary amino groups such as chitosan may require further optimization.

### 3.2 HAMA Purification and Characterization

HAMA is usually isolated by precipitation in ethanol and filtration. Other means of purification, such as dialysis, freeze-drying, or centrifugation, were explored in this work. After the reaction, the crude can be either precipitated in ethanol or dialyzed. If precipitated in the first instance, the polymer can be recovered by filtration or centrifugation. The second option is especially convenient when working with small batches. In the case of purifying HAMA by dialysis, the final product can be obtained by precipitation in ethanol or freeze-drying. While dialysis will lead to a product of a high purity, it is time- (2–3 days) and resources-consuming (membranes). While filtration and centrifugation produced a white solid, freeze-drying led to a “cotton wool” type of material, which was easier to handle. However, like dialysis, lyophilization is also time- and resources-consuming. Additionally, since freeze-drying was reported to degrade HA,^42^ this means of purification was avoided whenever possible. The precipitation followed by centrifugation strategy was particularly useful for low molecular weight HA, which led to a small particle size during precipitation, and dramatically increased the filtration time from minutes to hours. Recovery by precipitation and centrifugation was the preferred method for the screening reactions although some traces of unreacted MA were often found in the ^1^H NMR spectra. However, as can be seen in Figure 2(D), the signals of the impurity do not interfere with the broad peaks of the methacrylic group covalently linked to the polymer. Nor does the presence of traces of the impurity affect the conclusions from the screening experiments. The ^1^H NMR spectrum of a fully purified sample is shown in Figure S1. Regarding filtration, better recovery yields were obtained when polycarbonate membrane filters were used since HAMA hardens on regular filter paper, making it difficult to retrieve it.

As previously reported in the literature, the DM was estimated by integration of the ^1^H NMR spectra. Due to the high viscosity of HA solutions at low concentrations, obtaining good-quality NMR spectra can be challenging, in particular for high molecular weight HA.^43^ The ^1^H NMR spectra also showed multiple peaks (Figure S1) for the methacrylic protons indicating different methacrylation sites, especially when using low molecular weight HA. Even though the most favorable position for esterification is the primary alcohol (C-6), Seidlits et al. previously reported a DM of 160%,^33^ which is consistent with methacrylation taking place at multiple sites.

### 3.3 Characterization of the HAMA Hydrogels

The hydrogels were prepared by photo-cross-linking in PBS using I2959 as an initiator. Irgacure is a widely used photoinitiator due to its high biocompatibility, initiation by 365 nm UV radiation of low intensity and short irradiation time requirement.^44^

**Figure 3:**
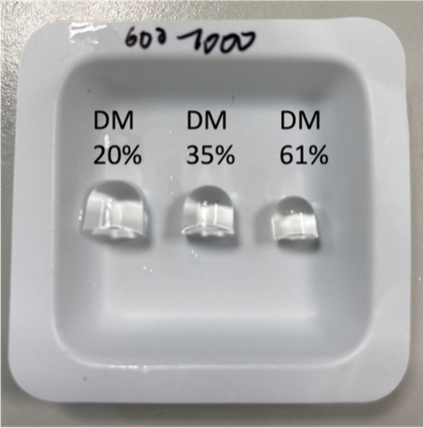
Hydrogels of 600–100 kDa HA after swelling for 24 h in PBS. The hydrogels were prepared from HAMA solutions at a concentration of 1%.

**Figure 4:**
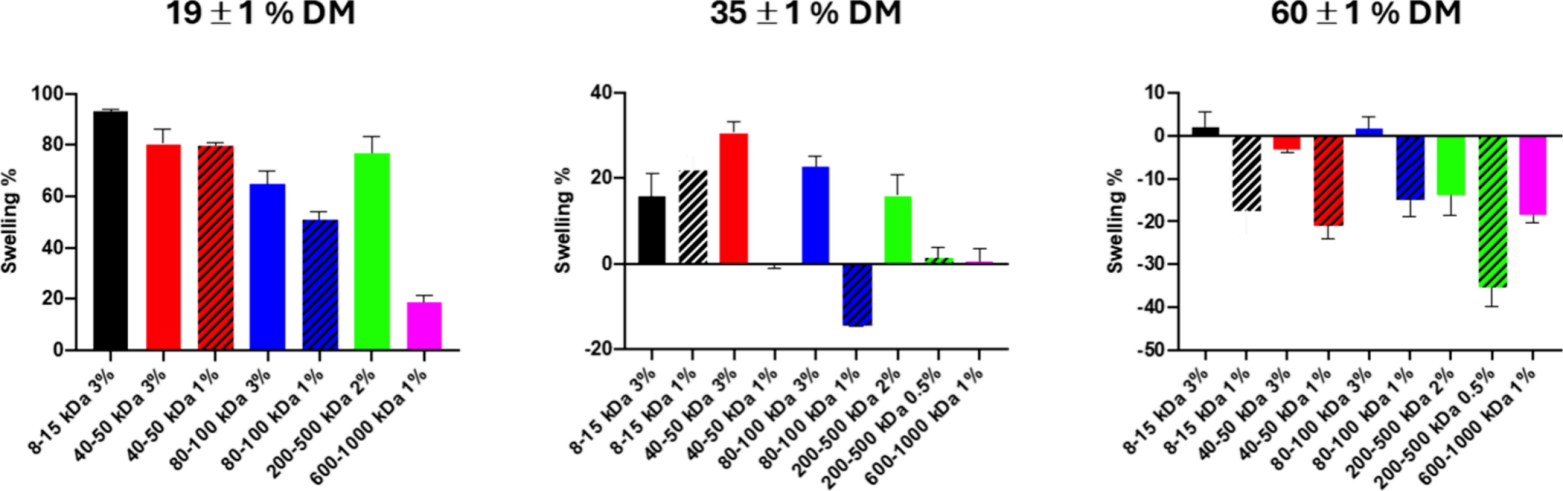
Degree of swelling of HAMA with different DMs (19, 35, and 60%). Columns with different colors/patterns represent HAMA of different molecular weights (8–15, 40–50, 80–100, 200–500, and 600–1000 kDa) and different concentrations of the HAMA solution subjected to photo-cross-linking (3, 2, 1, and 0.5 wt % in PBS). The degree of swelling was calculated as [(*W*_24_ – *W*_0_)/*W*_0_] × 100, where *W*_0_ and *W*_24_ are the weight before and after 24 h incubation in PBS, respectively. Samples were analyzed in triplicate.

The ability to retain its shape under physiological conditions is an important property of a hydrogel. Therefore, the swelling of the hydrogels caused by water uptake was assessed. The influence of the following parameters on the swelling behavior was studied; concentration of the HAMA solution subjected to cross-linking (0.5–3% wt in PBS), DM, and the molecular weight of HAMA. Figure 3 shows gels of 600–1000 kDa HAMA with different DMs after swelling for 24 h in PBS. The degree of swelling for different molecular weights, concentrations, and DMs is plotted in Figure 4. It was observed that lower DMs led to a higher swelling degree, while gels presenting higher functionalization displayed loss of water. The concentration of HAMA in the cross-linking solution also had an impact on the swelling. At higher concentrations (2 or 3%) and low DM, hydrogels displayed a strong positive swelling, while at lower concentrations and high DM, their loss of water was more significant. HAMA with a low molecular weight and DM gives viscous liquids rather than gels. Photo-cross-linking of a 1% wt solution of 8–15 kDa HAMA with a 19% DM did not result in an adequate hydrogel to perform swelling studies. The same was the case for a 0.5% wt solution of 200–500 kDa HAMA with a 17% DM. The molecular weight of the polymer seems to influence the degree of swelling but only at low DM (20%). Under the same conditions, low molecular weight HA presented higher PBS uptake; however, this influence seems to be mitigated at higher DMs.

As there are three variables (DM, molecular weight, and concentration of the solution, the hydrogel was prepared from), it is difficult to rationalize a trend in the swelling behavior. Hydrogels prepared using 80–100 kDa HAMA with a DM of around 35% swell when prepared from a 3 wt % solution and lose water when prepared from a 1 wt % solution. Shrinkage is generally observed when the DM is high (Figure 4). Oudshoorn et al. studied HAMA hydrogels with low degrees of substitution (<30%) and did not report any negative swelling.^32^ Shrinking was observed for HAMA hydrogels loaded with cardiomyocytes^45^ and mesenchymal stem cells (MSCs).^46^ However, this was not related to the gel itself but was attributed to the MSCs exerting a contractile traction force under chondrogenic induction.^46^ It seems that in our case, during the rapid photoinduced gelation, more water becomes entrapped than the amount corresponding to the equilibrium content.

**Figure 5:**
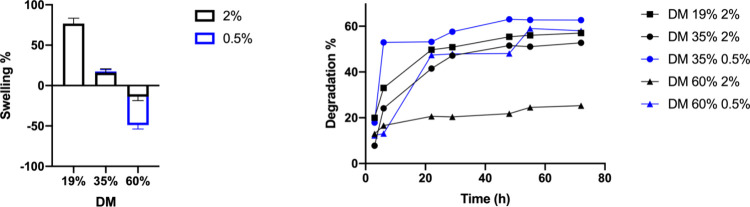
Swelling and enzymatic degradation (hyaluronidase) results were performed on HAMA gels (200–500 kDa) with 19, 35, and 60% DM. Gels were prepared from PBS solutions containing 2 or 0.5% HAMA and 0.3% I2959.

Next, we assessed the stability of the hydrogels toward enzymatic degradation. Hydrogels presenting fast or moderate enzymatic degradation have been found to be useful in the fields of drug delivery,^17^ while more resilient gels are beneficial for tissue engineering applications.^47^ It was observed that hydrogels presenting higher swelling degrees also displayed similar trends during enzymatic degradation in the presence of hyaluronidase. Gels prepared from solutions containing a lower concentration of HAMA degraded faster and to a larger extent (Figures 5 and S4–S8). As for the swelling, the DM had an impact during the enzymatic digestion of the hydrogels. Hydrogels presenting lower levels of cross-linking, either due to a low DM or low concentration of the polymer in the cross-linking solution, presented more and faster degradation in the first 12 h than those having higher cross-linking densities (Figures S4–S8). Tsanaktsidou et al. reported a similar behavior, finding a correlation between the degree of substitution and swelling, and degradation in their work.^48^

The hydrogels were further characterized by rheology. Frequency sweep measurements in the linear viscoelastic region (Figures S2 and S3) showed the expected features of viscoelastic solids with *G*′ (elastic modulus) > *G″* (viscous modulus).^49^*G*′ is a measure of the hydrogel’s stiffness and ability to retain its shape. The resistance to external forces is an important property with a view to applications. To be able to be used as a scaffold for cell growth, gels have to be rigid enough while keeping appropriate mechanical stiffness.^1−5^

The *G*′ values for the different hydrogels determined at a shear stress of 10 Pa and a frequency of 1 Hz are presented in Table 3. For hydrogels prepared from a 3% 8–15 kDa HAMA solution, *G*′ is 6.8, 86, and 249.7 kPa for a DM of 20, 35, and 60%, respectively. Likewise, gels of 40–50 and 80–100 kDa HAMA become stiffer with increasing DM. 200–500 kDa and 600–1000 kDa of HAMA, however, do not show a clear trend. According to classical statistical mechanics theory, the elastic modulus is directly proportional to the cross-link density.^50^*G*′ of the gels prepared from 8 to 15, 40 to 50, and 80 to 100 kDa HAMA at 3 wt % concentration increases linearly with the DM. This trend is not followed in the case of 200–500 and 600–1000 kDa HAMA. In all experiments, the same photoinitiator concentration was used. A possible explanation for the irregular trend of the high MW gels is the effect of the molecular weight on the viscosity of the HAMA solution.^51^ A high solution viscosity may affect the efficiency of the photoreaction, resulting in fewer photoreacted methacrylate residues at a given initiator concentration.

**Table 3:** *G*′ (kPa) and *G″* (kPa) Determined at a Shear Stress of 10 Pa and a Frequency of 1 Hz

M.W.	DM (%)	concentration (%)	*G*′ (kPa)	*G*″ (kPa)
8–15	19 ± 1	3	6.8 ± 0.3	2.2 ± 0.0
	35 ± 1	3	86 ± 2.3	14.0 ± 0.3
	35 ± 1	1	11.5 ± 0.3	2.1 ± 0.0
	60 ± 1	3	249.7 ± 10.0	6.1 ± 0.1
	60 ± 1	1	21.2 ± 0.6	4.9 ± 0.1
40–50	19 ± 1	3	24.0 ± 1.4	10.6 ± 0.3
	19 ± 1	1	3.6 ± 0.1	1.1 ± 0.0
	35 ± 1	3	62.4 ± 2.6	14.8 ± 0.1
	35 ± 1	1	10.8 ± 0.4	4.0 ± 0.1
	60 ± 1	3	102.0 ± 3.4	29.8 ± 0.2
	60 ± 1	1	49.6 ± 1.0	10.4 ± 0.0
80–100	19 ± 1	3	17.2 ± 1.1	14.9 ± 0.4
	19 ± 1	1	6.0 ± 0.2	2.1 ± 0.0
	35 ± 1	3	25.9 ± 0.7	18.0 ± 0.2
	35 ± 1	1	7.9 ± 0.6	3.9 ± 0.2
	60 ± 1	3	44.1 ± 0.9	21.8 ± 0.1
	60 ± 1	1	32.3 ± 0.5	10.7 ± 0.2
200–500	19 ± 1	2	35.3 ± 1.5	25.0 ± 0.2
	35 ± 1	2	25.0 ± 0.8	12.6 ± 0.3
	35 ± 1	0.5	6.8 ± 0.3	2.3 ± 0.0
	60 ± 1	2	14.0 ± 0.4	9.4 ± 0.2
	60 ± 1	0.5	11.1 ± 0.4	3.5 ± 0.0
600–1000	20 ± 1	1	7.3 ± 0.3	4.5 ± 0.1
	35 ± 1	1	28.3 ± 0.9	10.0 ± 0.0
	60 ± 1	1	10.8 ± 0.5	5.1 ± 0.0

When gels with the same molecular weight and DM are compared, a higher concentration of the cross-linking solution gives a stiffer gel. Both, the DM and the concentration of the solution the gel is prepared from affect the cross-linking density and the observed trends in *G*′ are as expected. The hydrogel with the highest mechanical resistance is formed when a 3% solution of 8–15 kDa HAMA with a DM of 60% is photo-cross-linked. This gel also has a slow enzymatic degradation rate (see above). In summary, the rheological data show that our improved synthesis provides easy access to hydrogels with distinct mechanical properties.

## 4 Conclusions

In this work, we developed a new and robust protocol for the preparation of HAMA with highly reproducible DMs. The method is scalable up to gram quantities and applicable to a wide range of molecular weights. By using a titration strategy, it is possible to obtain HAMA with a predefined DM of 19 ± 1, 35 ± 1, or 60 ± 1%, simply by adjusting the number of equivalents of base added. Compared with previous procedures, we could reduce the reaction time from 24 to 3 h. While literature methods typically use a 20-fold excess of MA to achieve high DMs, we could obtain HAMA with a DM of 60% by using a 5-fold excess of MA. By using a lower excess of MA and avoiding the use of buffers, the purification was also simplified. In contrast to previous methods that used organic solvents as cosolvents to increase the DM above 50%,^26,32^ the methacrylation reaction was performed in water as the sole solvent, thus presenting a greener alternative. Rheology and enzymatic degradation studies after photo-cross-linking confirmed that the new synthesis protocol developed in this work is a convenient method to tailor the properties of HA-based hydrogels. Besides HAMA, hybrid hydrogels of HAMA and a second biopolymer are widely investigated.^52^ The synthesis of HAMA with an adjustable and reproducible DM also offers new opportunities in this field.

## Supporting Information Available

Tables of HAMA screening reactions, ^1^H NMR spectrum of HAMA, rheological data, plots of swelling and enzymatic degradation data of HAMA gels (PDF)

## Abbreviations

HA: hyaluronic acid
HAMA: hyaluronic acid methacrylated
