# Supplementary material for: Improved and Highly Reproducible Synthesis of Methacrylated Hyaluronic Acid with Tailored Degrees of Substitution
Source: ACS Omega. 2024 Jun 6;9(24):25914–21. doi: 10.1021/acsomega.4c00372 (PMC11191076; doi:10.1021/acsomega.4c00372)
Supplement: Supplementary file 1 — ao4c00372_si_001.pdf [file ao4c00372_si_001.pdf]

Supplementary Information

Improved and highly reproducible synthesis of methacrylated hyaluronic acid with tailored degrees of substitution

Marta Pérez-Lloret, Andrea Erxleben*
School of Biological and Chemical Sciences, University of Galway, Ireland

Table of Contents

TABLES OF HAMA SCREENING REACTIONS

Table S1. Summary of the conditions for the screening reactions of HA (80-100 kDa) with 5 eq of MA under stirring in dark conditions. The pH was corrected prior to MA additions.

Table S2. Summary of the conditions for the screening reactions of HA (80-100 kDa) with 5 or 8.3 eq of MA under stirring in dark conditions. The pH was corrected prior to MA additions.

Table S3. Summary of the conditions for the screening reactions of HA with 1, 5 or 10 eq of MA under stirring in dark conditions. NaOH (1 M, 1 equiv./addition) was added every 30 minutes; 4 additions in total.

Table S4. Summary of the conditions for the screening reactions of HA with 1, 5 or 10 eq of MA under stirring in dark conditions. NaOH (1 M, 0.5 equiv./addition) was added every 30 minutes; 4 additions in total.

Table S5. Summary of the conditions for the screening reactions of HA with 1, 5 or 10 eq of MA under stirring in dark conditions. NaOH (1 M, 2 equiv./addition) was added every 30 minutes; 4 additions in total.

Table S6. Summary of the conditions for the screening reactions of HA (80-100 kDa) with 1, 2, 3, 4 or 5 eq of MA under stirring in dark conditions at 4ºC. NaOH (1 M, 1 equiv./addition) was added every 15 or 30 minutes; 4, 5, 6, 7 or 8 additions in total.

^{1}H NMR SPECTRUM

Figure S1. ^{1}H NMR spectrum (D_2O, 500 MHz) of HAMA (8-15 kDa).

RHEOLOGICAL STUDIES

Figure S2. Rheological response of HAMA gels of different DM, concentration and polymeric weight (A: 8-15 kDa, B: 40-50 kDa and C: 80-100 kDa).

Figure S3. Rheological response of HAMA gels of different DM, concentration and polymeric weight (A: 200-500 kDa and B: 600-1000 kDa).

SWELLING AND ENZYMATIC DEGRADATION

Figure S4. Swelling and enzymatic degradation of HAMA gels (8-15 kDa)

Figure S5. Swelling and enzymatic degradation of HAMA gels (40-50 kDa).

Figure S6. Swelling and enzymatic degradation of HAMA gels (80-100 kDa)

Figure S7. Swelling and enzymatic degradation of HAMA gels (200-500 kDa)

Figure S8. Swelling and enzymatic degradation of HAMA gels (600-1000 kDa)

Table S1. *Summary of the conditions for the screening reactions of HA (80-100 kDa) with 5 equiv. of MA under stirring in dark conditions. The pH was corrected prior to MA additions.*

**Sample nº**	**Solvent**	**T (°C)**	**Buffer**	**Titration (additions)**	**Time**	**HA concentration**	**MA V (5 eq)**	**DM %**
HA-MA_Sc_001	H_2O	0			24h	20 mg in 2 mL	40 μL	x
HA-MA_Sc_002	H_2O	30			24h	20 mg in 2 mL	40 μL	3-4
HA-MA_Sc_003	H_2O	0		6 every 30 min	3h	30 mg in 3 mL	60 μL (6x10 μL)	37-41
HA-MA_Sc_004	H_2O	30		6 every 30 min	3h	30 mg in 3 mL	60 μL (6x10 μL)	26-29
HA-MA_Sc_005	H_2O	0	PBS		24h	20 mg in 2 mL	40 μL	X
HA-MA_Sc_006	H_2O	30	PBS		24h	20 mg in 2 mL	40 μL	2-3
HA-MA_Sc_007	H_2O	0	PBS	6 every 30 min	3h	30 mg in 3 mL	60 μL (6x10 μL)	20
HA-MA_Sc_008	H_2O	30	PBS	6 every 30 min	3h	30 mg in 3 mL	60 μL (6x10 μL)	15-20
HA-MA_Sc_009	H_2O	0	CB		24h	20 mg in 2 mL	40 μL	11-16
HA-MA_Sc_010	H_2O	30	CB		24h	20 mg in 2 mL	40 μL	22-26
HA-MA_Sc_011	H_2O	0	CB	6 every 30 min	3h	30 mg in 3 mL	60 μL (6x10 μL)	22
HA-MA_Sc_012	H_2O	30	CB	6 every 30 min	3h	30 mg in 3 mL	60 μL (6x10 μL)	29
HA-MA_Sc_013	DMSO	30			24h	20 mg in 2 mL	40 μL	X
HA-MA_Sc_014	H_2O/DMSO 1:1	30			24h	20 mg in 2 mL	40 μL	2-3
HA-MA_Sc_015	H_2O/DMSO 1:1	30	PBS		24h	20 mg in 2 mL	40 μL	10
HA-MA_Sc_016	H_2O/DMSO 1:1	30	PBS	6 every 30 min	3h	30 mg in 3 mL	60 μL (6x10 μL)	X
HA-MA_Sc_017	H_2O/DMSO 1:1	30	CB		24h	20 mg in 2 mL	40 μL	5-7
HA-MA_Sc_018	H_2O/DMSO 1:1	30	CB	6 every 30 min	3h	30 mg in 3 mL	60 μL (6x10 μL)	28-33

Table S2. *Summary of the conditions for the screening reactions of HA (80-100 kDa) with 5 or 8.3 equiv. of MA under stirring in dark conditions. The pH was corrected prior to MA additions.*

**Sample nº**	**Solvent**	**T (°C)**	**Buffer**	**Titration (additions)**	**Time**	**HA concentration**	**MA V**	**MA eq**	**DM**
HA-MA_Sc_020	H_2O	0		6 every 30 min	3h	30 mg in 3 mL	60 μL (6x10 μL)	5	37-41
HA-MA_Sc_021	H_2O	30		6 every 30 min	3h	30 mg in 3 mL	60 μL (6x10 μL)	5	26-29
HA-MA_Sc_022	H_2O	0		6 every 1h	6h	30 mg in 3 mL	60 μL (6x10 μL)	5	35-38
HA-MA_Sc_023	H_2O	30		6 every 1h	6h	30 mg in 3 mL	60 μL (6x10 μL)	5	35-35
HA-MA_Sc_024	H_2O	0		10 every 30 min	5h	30 mg in 3 mL	100 μL (10x10 μL)	8.3	25-28
HA-MA_Sc_025	H_2O	30		10 every 30 min	5h	30 mg in 3 mL	100 μL (10x10 μL)	8.3	22-30
HA-MA_Sc_026	H_2O	0	PBS	6 every 30 min	3h	30 mg in 3 mL	60 μL (6x10 μL)	5	19-20
HA-MA_Sc_027	H_2O	30	PBS	6 every 30 min	3h	30 mg in 3 mL	60 μL (6x10 μL)	5	15-20
HA-MA_Sc_028	H_2O	0	PBS	6 every 1h	6h	30 mg in 3 mL	60 μL (6x10 μL)	5	32-34
HA-MA_Sc_029	H_2O	30	PBS	6 every 1h	6h	30 mg in 3 mL	60 μL (6x10 μL)	5	6-9
HA-MA_Sc_030	H_2O	0	PBS	10 every 30 min	5h	30 mg in 3 mL	100 μL (10x10 μL)	8.3	48-52
HA-MA_Sc_031	H_2O	30	PBS	10 every 30 min	5h	30 mg in 3 mL	100 μL (10x10 μL)	8.3	34-37
HA-MA_Sc_032	H_2O	0	CB	6 every 30 min	3h	30 mg in 3 mL	60 μL (6x10 μL)	5	21-23
HA-MA_Sc_033	H_2O	30	CB	6 every 30 min	3h	30 mg in 3 mL	60 μL (6x10 μL)	5	28-29
HA-MA_Sc_034	H_2O	0	CB	6 every 1h	6h	30 mg in 3 mL	60 μL (6x10 μL)	5	37
HA-MA_Sc_035	H_2O	30	CB	6 every 1h	6h	30 mg in 3 mL	60 μL (6x10 μL)	5	26-27
HA-MA_Sc_036	H_2O	0	CB	10 every 30 min	5h	30 mg in 3 mL	100 μL (10x10 μL)	8.3	60
HA-MA_Sc_037	H_2O	30	CB	10 every 30 min	5h	30 mg in 3 mL	100 μL (10x10 μL)	8.3	40-41

Table S3. *Summary of the conditions for the screening reactions of HA with 1, 5 or 10 equiv. of MA under stirring in dark conditions. NaOH (1M, 1 equiv./addition) was added every 30 minutes; 4 additions in total.*

**Sample nº**	**Solvent**	**T (°C)**	**HA concentration**	**MW**	**MA eq**	**MA V**	**NaOH 1 M V/addition**	**DM**
HA-MA_Sc_120	H_2O	0	20 mg in 2 mL	8-15 kDa	1	8 μL	49.6 μL	X
HA-MA_Sc_121	H_2O	RT	20 mg in 2 mL	8-15 kDa	1	8 μL	49.6 μL	X
HA-MA_Sc_122	H_2O	0	20 mg in 2 mL	40-50 kDa	1	8 μL	49.6 μL	X
HA-MA_Sc_123	H_2O	RT	20 mg in 2 mL	40-50 kDa	1	8 μL	49.6 μL	X
HA-MA_Sc_124	H_2O	0	20 mg in 2 mL	80-100 kDa	1	8 μL	49.6 μL	X
HA-MA_Sc_125	H_2O	RT	20 mg in 2 mL	80-100 kDa	1	8 μL	49.6 μL	X
HA-MA_Sc_126	H_2O	0	20 mg in 2 mL	8-15 kDa	5	40 μL	49.6 μL	35
HA-MA_Sc_127	H_2O	RT	20 mg in 2 mL	8-15 kDa	5	40 μL	49.6 μL	19
HA-MA_Sc_128	H_2O	0	20 mg in 2 mL	40-50 kDa	5	40 μL	49.6 μL	35
HA-MA_Sc_129	H_2O	RT	20 mg in 2 mL	40-50 kDa	5	40 μL	49.6 μL	14
HA-MA_Sc_130	H_2O	0	20 mg in 2 mL	80-100 kDa	5	40 μL	49.6 μL	33
HA-MA_Sc_131	H_2O	RT	20 mg in 2 mL	80-100 kDa	5	40 μL	49.6 μL	12
HA-MA_Sc_132	H_2O	0	20 mg in 2 mL	8-15 kDa	10	80 μL	49.6 μL	34
HA-MA_Sc_133	H_2O	RT	20 mg in 2 mL	8-15 kDa	10	80 μL	49.6 μL	15
HA-MA_Sc_134	H_2O	0	20 mg in 2 mL	40-50 kDa	10	80 μL	49.6 μL	33
HA-MA_Sc_135	H_2O	RT	20 mg in 2 mL	40-50 kDa	10	80 μL	49.6 μL	15
HA-MA_Sc_136	H_2O	0	20 mg in 2 mL	80-100 kDa	10	80 μL	49.6 μL	22
HA-MA_Sc_137	H_2O	RT	20 mg in 2 mL	80-100 kDa	10	80 μL	49.6 μL	11

Table S4. *Summary of the conditions for the screening reactions of HA with 1, 5 or 10 equiv. of MA under stirring in dark conditions. NaOH (1 M, 0.5 equiv./addition) was added every 30 minutes; 4 additions in total.*

Sample nº	Solvent	T (°C)	HA concentration	MW	MA eq	MA	NaOH 1 M, V/addition	DM
HA-MA_Sc_140	H_2O	0	20 mg in 2 mL	8-15 kDa	1	8 μL	24.8 μL	X
HA-MA_Sc_141	H_2O	RT	20 mg in 2 mL	8-15 kDa	1	8 μL	24.8 μL	2
HA-MA_Sc_142	H_2O	0	20 mg in 2 mL	40-50 kDa	1	8 μL	24.8 μL	3
HA-MA_Sc_143	H_2O	RT	20 mg in 2 mL	40-50 kDa	1	8 μL	24.8 μL	X
HA-MA_Sc_144	H_2O	0	20 mg in 2 mL	80-100 kDa	1	8 μL	24.8 μL	2
HA-MA_Sc_145	H_2O	RT	20 mg in 2 mL	80-100 kDa	1	8 μL	24.8 μL	X
HA-MA_Sc_146	H_2O	0	20 mg in 2 mL	8-15 kDa	5	40 μL	24.8 μL	15
HA-MA_Sc_147	H_2O	RT	20 mg in 2 mL	8-15 kDa	5	40 μL	24.8 μL	5
HA-MA_Sc_148	H_2O	0	20 mg in 2 mL	40-50 kDa	5	40 μL	24.8 μL	15
HA-MA_Sc_149	H_2O	RT	20 mg in 2 mL	40-50 kDa	5	40 μL	24.8 μL	2
HA-MA_Sc_150	H_2O	0	20 mg in 2 mL	80-100 kDa	5	40 μL	24.8 μL	15
HA-MA_Sc_151	H_2O	RT	20 mg in 2 mL	80-100 kDa	5	40 μL	24.8 μL	X
HA-MA_Sc_152	H_2O	0	20 mg in 2 mL	8-15 kDa	10	80 μL	24.8 μL	11
HA-MA_Sc_153	H_2O	RT	20 mg in 2 mL	8-15 kDa	10	80 μL	24.8 μL	6
HA-MA_Sc_154	H_2O	0	20 mg in 2 mL	40-50 kDa	10	80 μL	24.8 μL	13
HA-MA_Sc_155	H_2O	RT	20 mg in 2 mL	40-50 kDa	10	80 μL	24.8 μL	2
HA-MA_Sc_156	H_2O	0	20 mg in 2 mL	80-100 kDa	10	80 μL	24.8 μL	X
HA-MA_Sc_157	H_2O	RT	20 mg in 2 mL	80-100 kDa	10	80 μL	24.8 μL	X

Table S5. *Summary of the conditions for the screening reactions of HA with 1, 5 or 10 equiv. of MA under stirring in dark conditions. NaOH (1 M, 2 equiv./addition) was added every 30 minutes; 4 additions in total.*

**Sample nº**	**Solvent**	**T (°C)**	**HA concentration**	**MW**	**MA eq**	**MA**	**NaOH 1 M V/addition**	**DM**
HA-MA_Sc_160	H_2O	0	20 mg in 2 mL	8-15 kDa	1	8 μL	99.2 μL	X
HA-MA_Sc_161	H_2O	RT	20 mg in 2 mL	8-15 kDa	1	8 μL	99.2 μL	3
HA-MA_Sc_162	H_2O	0	20 mg in 2 mL	40-50 kDa	1	8 μL	99.2 μL	X
HA-MA_Sc_163	H_2O	RT	20 mg in 2 mL	40-50 kDa	1	8 μL	99.2 μL	X
HA-MA_Sc_164	H_2O	0	20 mg in 2 mL	80-100 kDa	1	8 μL	99.2 μL	X
HA-MA_Sc_165	H_2O	RT	20 mg in 2 mL	80-100 kDa	1	8 μL	99.2 μL	X
HA-MA_Sc_166	H_2O	0	20 mg in 2 mL	8-15 kDa	5	40 μL	99.2 μL	78
HA-MA_Sc_167	H_2O	RT	20 mg in 2 mL	8-15 kDa	5	40 μL	99.2 μL	45
HA-MA_Sc_168	H_2O	0	20 mg in 2 mL	40-50 kDa	5	40 μL	99.2 μL	80
HA-MA_Sc_169	H_2O	RT	20 mg in 2 mL	40-50 kDa	5	40 μL	99.2 μL	41
HA-MA_Sc_170	H_2O	0	20 mg in 2 mL	80-100 kDa	5	40 μL	99.2 μL	73
HA-MA_Sc_171	H_2O	RT	20 mg in 2 mL	80-100 kDa	5	40 μL	99.2 μL	35
HA-MA_Sc_172	H_2O	0	20 mg in 2 mL	8-15 kDa	10	80 μL	99.2 μL	70
HA-MA_Sc_173	H_2O	RT	20 mg in 2 mL	8-15 kDa	10	80 μL	99.2 μL	45
HA-MA_Sc_174	H_2O	0	20 mg in 2 mL	40-50 kDa	10	80 μL	99.2 μL	70
HA-MA_Sc_175	H_2O	RT	20 mg in 2 mL	40-50 kDa	10	80 μL	99.2 μL	46
HA-MA_Sc_176	H_2O	0	20 mg in 2 mL	80-100 kDa	10	80 μL	99.2 μL	76
HA-MA_Sc_177	H_2O	RT	20 mg in 2 mL	80-100 kDa	10	80 μL	99.2 μL	36

Table S6. *Summary of the conditions for the screening reactions of HA (80-100 kDa) with 1, 2, 3, 4 or 5 equiv. of MA under stirring in dark conditions at 4ºC. NaOH (1 M, 1 equiv./addition) was added every 15 or 30 minutes; 4, 5, 6, 7 or 8 additions in total.*

**Sample nº**	**Solvent**	**HA concentration**	**MA eq**	**MA**	**NaOH 1M V/addition**	**Nº of additions**	**Time /min**	**DM**
HA-MA_Sc_180	H_2O	20 mg in 2 mL	1	8 μL	49.6 μL	4	30	X
HA-MA_Sc_181	H_2O	20 mg in 2 mL	2	16 μL	49.6 μL	4	30	14
HA-MA_Sc_182	H_2O	20 mg in 2 mL	3	24 μL	49.6 μL	4	30	28
HA-MA_Sc_183	H_2O	20 mg in 2 mL	4	32 μL	49.6 μL	4	30	35
HA-MA_Sc_184	H_2O	20 mg in 2 mL	5	40 μL	49.6 μL	4	30	35
HA-MA_Sc_185	H_2O	20 mg in 2 mL	5	40 μL	49.6 μL	4	15	27
HA-MA_Sc_186	H_2O	20 mg in 2 mL	5	40 μL	49.6 μL	5	30	47
HA-MA_Sc_187	H_2O	20 mg in 2 mL	5	40 μL	49.6 μL	6	30	55
HA-MA_Sc_188	H_2O	20 mg in 2 mL	5	40 μL	49.6 μL	7	30	71
HA-MA_Sc_189	H_2O	20 mg in 2 mL	5	40 μL	49.6 μL	8	30	80

1. ^{1}H NMR spectrum

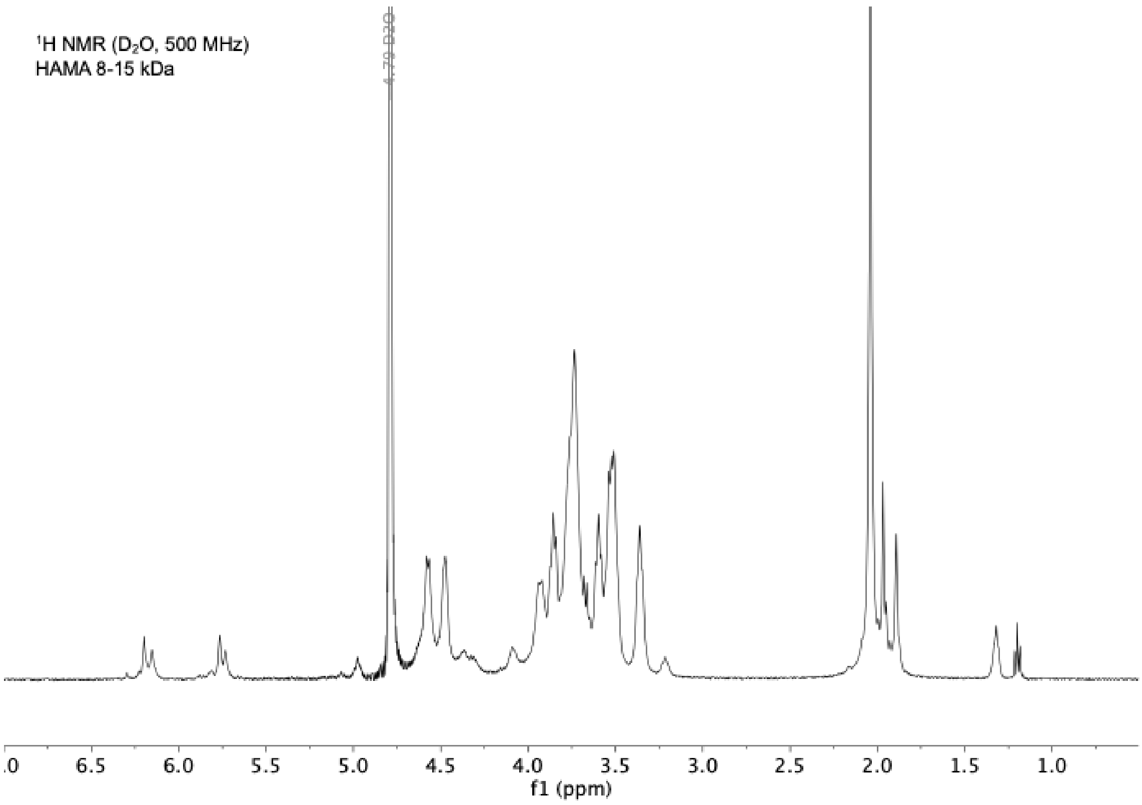

Figure S1. *^{1}H NMR spectrum (D_2O, 500 MHz) of HAMA (8-15 kDa).*

2. Rheological studies

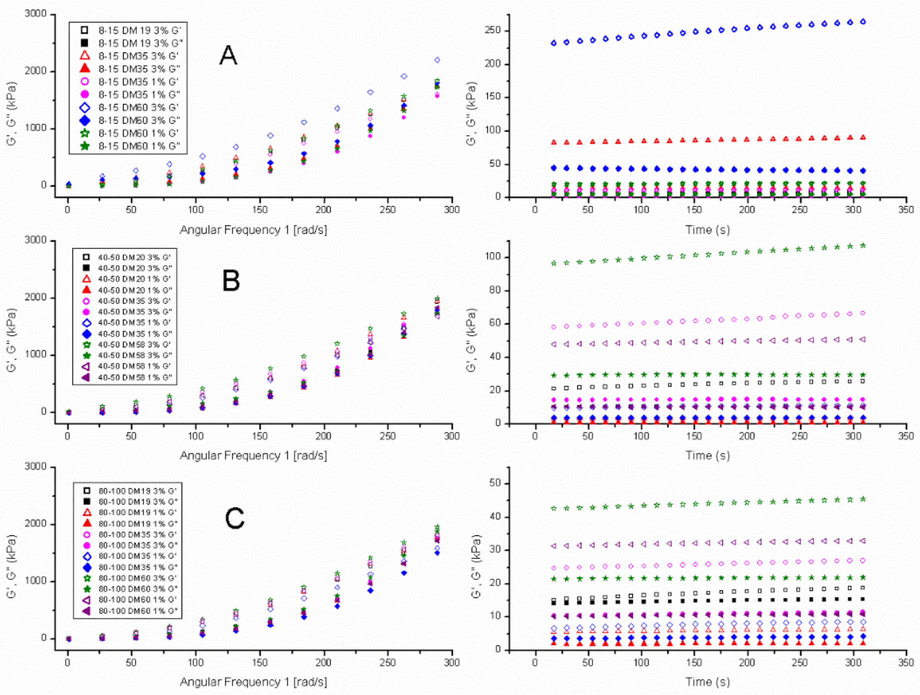

Figure S2. *Rheological response of HAMA gels of different DM, concentration and molecular weight (**A**: 8-15 kDa, **B**: 40-50 kDa and **C**: 80-100 kDa) to frequency sweep (left) at a constant shear strain (oscillating) of 10% and increasing frequency from 0.1 to 100 Hz, and amplitude sweep (right) constant shear strain (oscillating) of 10% and constant frequency at 1 Hz. Disk shaped hydrogels of 25 mm diameter and 10 mm height were prepared at different polymeric concentrations (3%w and 1%w) in PBS containing 0.3%w I2959.*

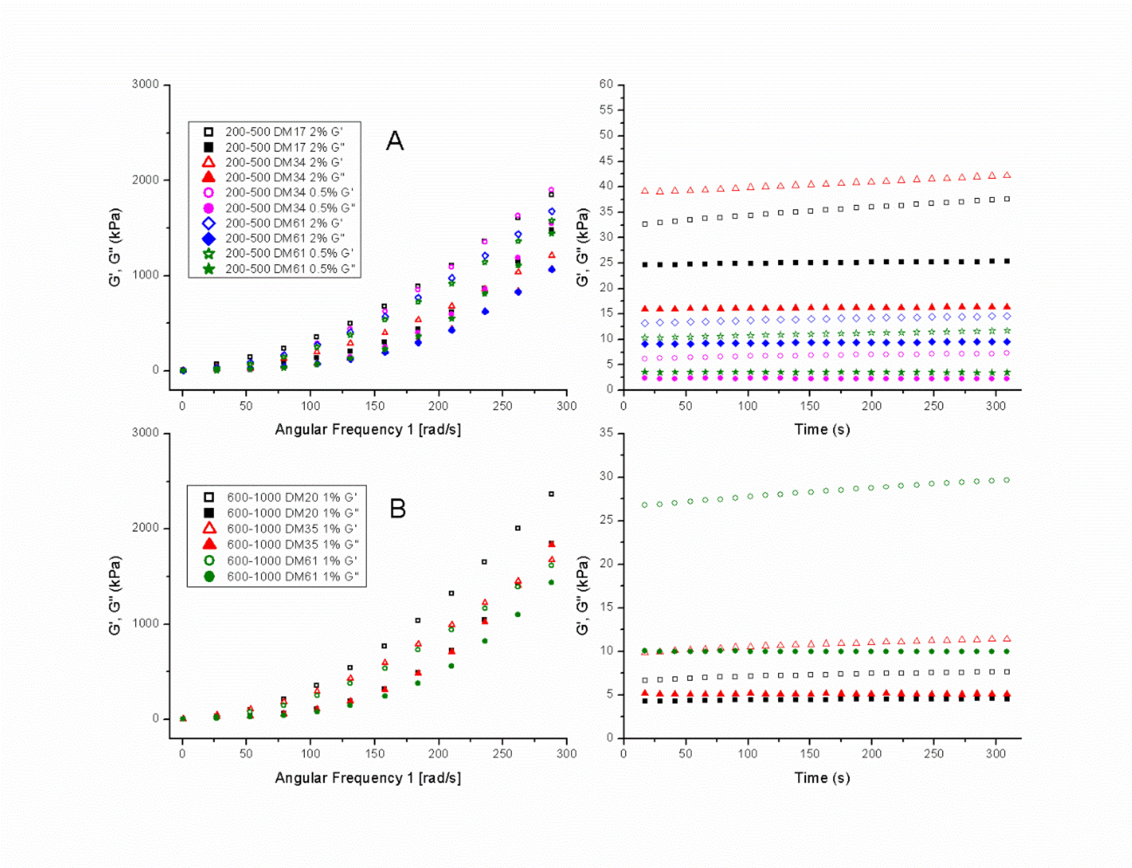

Figure S3. *Rheological response of HAMA gels of different DM, concentration and molecular weight (**A**: 200-500 kDa and **B**: 600-1000 kDa) to frequency sweep (left) at a constant shear strain (oscillating) of 10% and increasing frequency from 0.1 to 100 Hz, and amplitude sweep (right) constant shear strain (oscillating) of 10% and constant frequency at 1 Hz. Disk shaped hydrogels of 25 mm diameter and 10 mm height were prepared at different polymeric concentrations (2%w, 1%w and 0.5%w) in PBS containing 0.3%w I2959.*

3. Swelling and enzymatic degradation

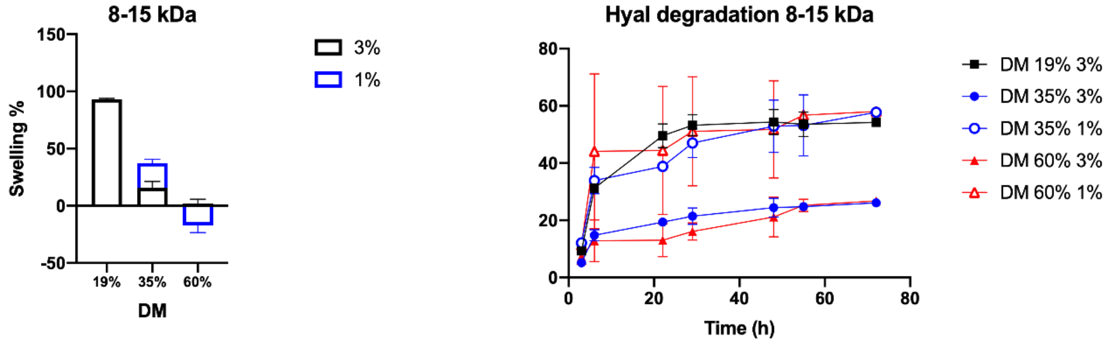

Figure S4. *Swelling and enzymatic degradation (100 U/mL hyaluronidase) performed on HAMA gels (8-15 kDa) with 19, 35 and 60% DM at 3% and 1% concentration in PBS containing 0.3%w of I2959.*

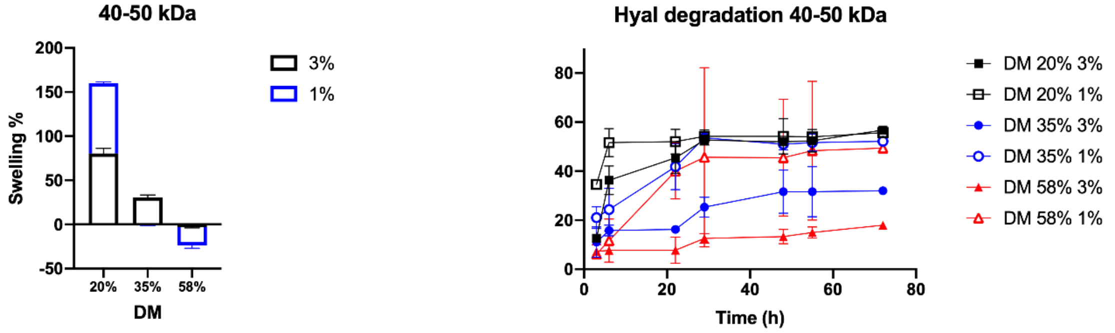

Figure S5. *Swelling and enzymatic degradation (100 U/mL hyaluronidase) performed on HAMA gels (40-50 kDa) with 20, 35 and 58% DM at 3% and 1% concentration in PBS containing 0.3%w of I2959.*

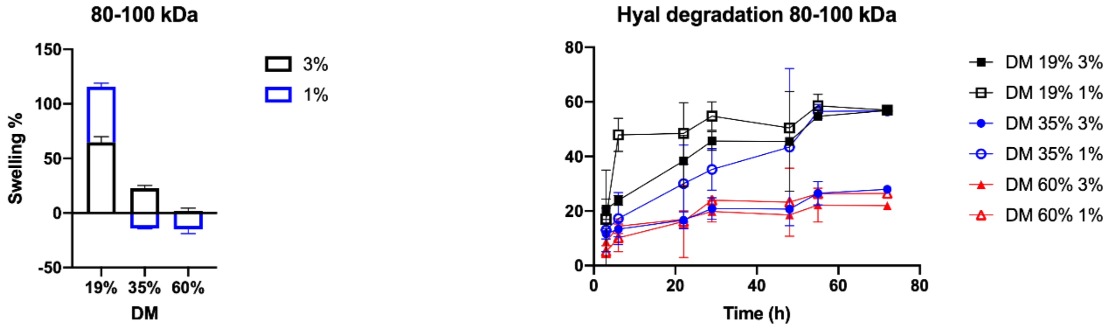

Figure S4. *Swelling and enzymatic degradation (100 U/mL hyaluronidase) performed on HAMA gels (80-100 kDa) with 19, 35 and 60% DM at 3% and 1% concentration in PBS containing 0.3%w of I2959.*

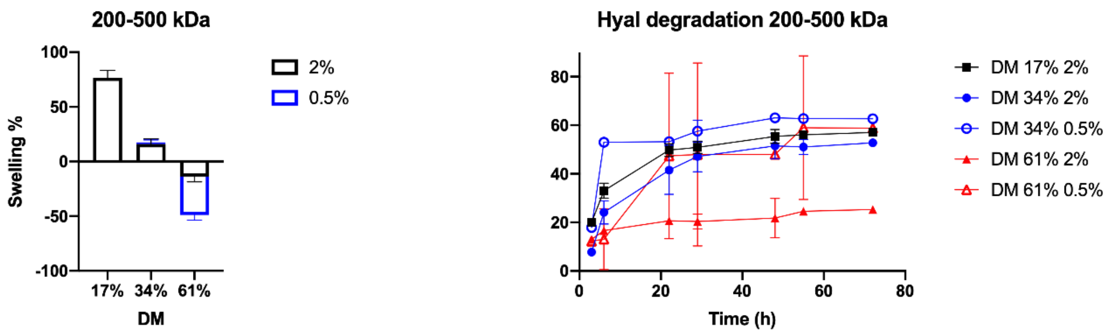

Figure S7. *Swelling and enzymatic degradation (100 U/mL hyaluronidase) performed on HAMA gels (200-500 kDa) with 17, 34 and 61% DM at 2% and 0.5% concentration in PBS containing 0.3%w of I2959.*

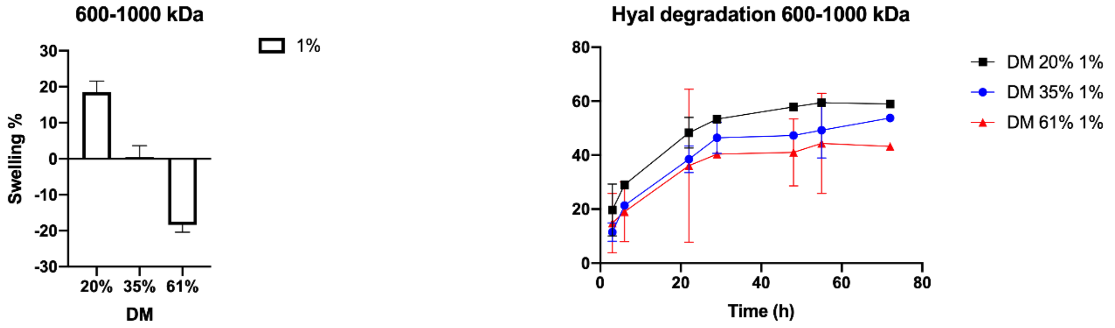

Figure S8. *Swelling and enzymatic degradation (100 U/mL hyaluronidase) performed on HAMA gels (200-500 kDa) with 20, 35 and 61% DM at 1% concentration in PBS containing 0.3%w of I2959.*
